# Providing cell phone numbers and e-mail addresses to patients: The patient’s perspective, a cross sectional study

**DOI:** 10.1186/2045-4015-1-32

**Published:** 2012-08-28

**Authors:** Roni Peleg, Elena Nazarenko

**Affiliations:** 1 Clalit Health Services, Southern District, and the Department of Family Medicine, Siaal Research Center, Faculty of Health Sciences, Ben-Gurion University, POB 653, Beer-Sheva, 84105, Israel

**Keywords:** Cell phone, E-mail, Patients, Physicians

## Abstract

**Background:**

Today patients can consult with their treating physician by cell phone or e-mail. These means of communication enhance the quality of medical care and increase patient satisfaction, but they can also impinge on physicians’ free time and their patient schedule while at work. The objective of this study is to assess the attitudes and practice of patients on obtaining the cell phone number or e-mail address of their physician for the purpose of medical consultation.

**Methods:**

Personal interviews with patients, 18 years of age or above, selected by random sampling from the roster of adults insured by Clalit Health Services, Southern Division. The total response rate was 41%. The questionnaire included questions on the attitude and practice of patients towards obtaining their physician’s cell phone number or e-mail address. Comparisons were performed using Chi-square tests to analyze statistically significant differences of categorical variables. Two-tailed *p* values less than 0.05 were considered statistically significant, with a power of 0.8.

**Results:**

The study sample included 200 patients with a mean age of 46.6 ± 17.1, of whom 110 were women (55%). Ninety-three (46.5%) responded that they would be very interested in obtaining their physician’s cell phone number, and an additional 83 (41.5%) would not object to obtaining it. Of the 171 patients (85.5%) who had e-mail addresses, 25 (14.6%) said they would be very interested in obtaining their physician’s e-mail address, 85 (49.7%) said they would not object to getting it, and 61 (35.7%) were not interested. In practice only one patient had requested the physician’s e-mail address and none actually had it.

**Conclusions:**

Patients favored cell phones over e-mail for consulting with their treating physicians. With new technologies such as cell phones and e-mail in common use, it is important to determine how they can be best used and how they should be integrated into the flow of clinical practice.

## Background

Israel has had a compulsory national health law since 1995. It provides for health care services to the entire population through health maintenance organizations (HMOs). In recent years there has been a growing competition between the various HMOs, which has led them to innovate and become more efficient to improve healthcare services and enhance patient satisfaction. The challenge is to improve the quality of care for patients and safeguard patients’ interests while meeting budget constraints and contributing to the overall efficiency of the healthcare system. These circumstances have increased the appeal of methods of communication such as providing patients with their physician’s personal cell phone number or e-mail address.

As stated in an article by McKinstry and colleagues in the BMJ, patients in the UK can turn to their physicians for medical advice by cell phone or e-mail with the aim of improving the quality of healthcare. Physicians use modern technology to provide care including consultation by means of cell phones and e-mail [1]. In several countries, requests for medical treatment outside of regular work hours are routinely triaged by telephone [2,3]. In the UK, a national survey showed that a growing number of patients want to establish contact with health care services by e-mail [4]. It is unclear whether these services are performed for a fee.

In an attempt to improve quality of care in the face of increasing workloads, clinicians use new methods to deliver medical care, including telephone consultations [5]. A number of studies have demonstrated that telephone consultations are shorter than face-to-face consultations [6,7] and may be time effective in managing chronic illness [8]. The appropriate use of telephone consultations can enhance access to medical care, and save time and travel for patients [1].

Patients who visit primary care clinics also contact their physicians frequently by phone [9]. The results of one study showed that 83.1% of telephone consults did not require a clinic visit as the problem was solved over the phone. It was possible to follow up on 58.2% of the cases through cell phone alone [10]. Most family physicians, who were surveyed in another study, defined telephone consultations as a reliable service [11].

Car and Sheikh, in a paper on e-mail consultations in health care state: “Electronic communication holds the promise for a revolution in health care for patients” [12]. A review of patient-physician communication found that patients were very happy about the possibility of communication with their physicians through e-mail. The use of e-mail for medical consultation was found to be convenient and practical, and no adverse effects were reported by physicians [13]. In another review of the role of e-mail in patient-provider communication, the authors stated that e-mail is changing the patient-physician relationship. e-mail communication can replace, at least in part, the traditional frontal form of patient-physician interactions. This expansion of means of communication in medicine necessitates an evaluation of their advantages and limitations. The appropriate use of e-mail can improve communication and serve as a central tool in the healthcare system [14].

A study that evaluated the experience of physicians who use e-mail for communication with patients found that the most important reasons for using e-mail, among those physicians who were satisfied with its use, were that it “saves time” (33%) and “helps deliver better care” (28%), compared with “the patients requested it” (80%) among those who were not satisfied with it [15].

To obtain optimal benefit from this form of service it is important that physicians understand its advantages and limitations. Although providing the telephone number [16] or e-mail address [15] to patients is easy and makes medical consultations simpler, it also can make the physician’s routine workload heavier, have a negative effect on the work environment, and even interfere with the physician’s free time [17].

Informal consultations between patients and physicians are commonplace as are informal consultations among physicians relating to their patients [18]. e-mail consultations are also a common means of communication for the same purpose [19]. The results of a study that was conducted in the Department of Family Medicine in Beer-Sheva, Israel [20], which assessed the provision of cell phone numbers and e-mail addresses to patients from the physician’s perspective, showed that the majority of physicians preferred to give their cell phone number rather than their e-mail address to patients.

Currently, the marketing departments of four HMOs are engaged in providing online medical services to the people insured. In an attempt to determine the policy of the four HMOs in Israel, we found that as of March 2012 guidelines and ethical and legal rules concerning providing medical services this way have not yet been determined. According to the current knowledge, there are no organizational guidelines that doctors give an e-mail address or personal mobile phone number to patients, and if they do it is at their own discretion.

Social media such as Facebook and Twitter serve as a prevailing means of communication today and could also serve as a tool for non-face-to-face medical consultations. In the present study we focused on consultations by means of cell phones and e-mail.

The primary objective of the present study was to assess the attitudes and practice of patients in relation to getting their physician’s cell phone number or e-mail address for medical consultations.

## Methods

**Figure 1:**
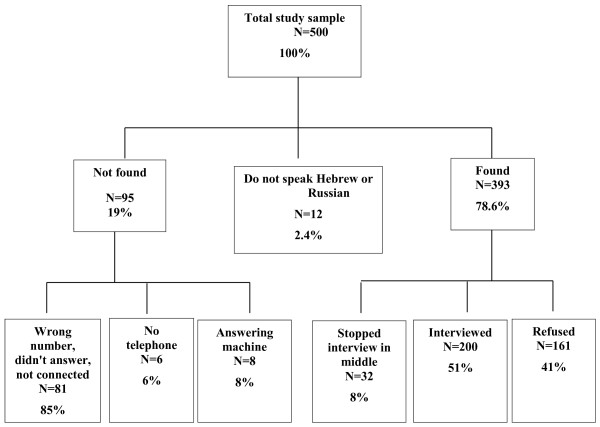
Flow diagram of participant recruitment to the study.

A sample of 500 adults, 18 years of age or above, was generated by random sampling from the roster of adults insured by the Clalit Health Services, Southern Division. The sampling was conducted by the Economics Department of the HMO. After providing informed consent the first 200 who agreed to participate in the study and spoke either Hebrew or Russian were interviewed by telephone by one of the investigators (EN) during the evening hours. In formulating the questionnaire we were helped by the experience gained from our previous study [20]. The questionnaire underwent revisions after a pilot study that included four physicians and ten non-physician patients who were not included in the study results. The questionnaire was not validated. Individuals who the interviewer felt did not understand the questionnaire or who were not cooperative were excluded. Data collection stopped when the interviewer reached the 200th patient (at patient number 485, 41% response rate). From the original list of 500 only 12 (2.4%) were not interviewed because they do not speak Hebrew or Russian. Details on the study sample are shown in Figure 1. 

The study was approved by The Helsinki Committee of Meir Medical Center (approval #148/09).

The first part of the questionnaire assessed patients’ attitudes regarding getting their physician’s cell phone number and e-mail address for medical consultations. The patients were instructed to answer the questionnaire only in relation to their family physician and not other medical consultants. It also referred to consultations with physicians only and not to general contact with practices, for example for administrative or other technical needs. The second part included the patient’s socio-demographic data.

The questionnaire was translated into Russian in order to include Russian speakers in the study. The translation was validated by the back-translation method.

Statistical analyses were performed using SPSS software, version 15 (SPSS, Chicago, IL, USA). Chi-square tests were used to analyze statistically significant differences of categorical variables. Two-tailed *p* values less than 0.05 were considered statistically significant, with a power of 0.8.

## Results

### Socio-demographic characteristics of the study population

The study population comprised 200 patients with a mean age of 46.6 ± 17.1, 110 (55%) of them women. In comparison, the percentage of women aged 18 years or above who are insured by the Clalit Health Services, Southern Division, is 52% and the mean age of all insured is 48 years. Ninety-four (47%) defined their health status as good to excellent. The socio-demographic characteristics of the study participants are shown in Table 1.

**Table 1:** Socio-demographic and health characteristics of the study population (N = 200)

**Variable**	**Result**
**Age in years (N = 118)**	
Mean ± SD	46.6 ± 17.1
Range	20–89
**Gender**	
Male	90 (45.0)
Female	110 (55.0)
**Family status**	
Single	55 (27.5)
Married	85 (42.5)
Divorced	31 (15.5)
Widowed	27 (13.5)
Separated	2 (1.0)
**Number of children**	
Mean ± SD	2.4 ± 2.3
Range	0–12
**Country of birth**	
Israel	79 (39.5)
Eastern Europe	58 (29.0)
Western Europe	34 (17/.0)
Other (North Africa, Asia, S. America)	29 (14.5)
**Years of education**	
Mean ± SD	11.5 ± 3.5
Range	1–18
**Present work status**	
Employed	126 (63.0)
Student	20 (10.0)
Unemployed	12 (6.0)
Retired	42 (21.0)
**How would you rate your health condition?**	
Very good to excellent	94 (47.0)
Fair to good	79 (39.5)
Poor	27 (13.5)
**Do you suffer from a chronic disease?**	
Yes	76 (38.0)
No	124 (62.0)

### Attitudes towards cell phone consultations with physicians

Ninety-three patients (46.5%) said that they would be very interested in getting their physician’s cell phone number, while 83 (41.5%) would not object to getting it, and only 24 (12%) would not be interested (Table 2).

**Table 2:** Attitudes to medical consultation through cell phones

**Question**	**N (%)**
**How do you feel about getting your physician’s cell phone number?**	
Very interested	93 (46.5)
Would not object	83 (41.5)
Not interested	24 (12.0)
**Getting my physician’s cell phone number could improve the relationship between us:**	
Agree	176 (88.0)
Do not agree	24 (12.0)
**Getting my physician’s cellphone number could improve my sense of security even if I don’t use it:**	
Agree	169 (84.5)
Do not agree	31 (14.5)
**The cell phone is an effective means of communication that could solve my problems:**	
Agree	102 (51.0)
Do not agree	98 (49.0)
**The cell phone can cut down on the number of clinic visits:**	
Agree	138 (69.0)
Do not agree	62 (31.0)
**The cell phone can reduce the number of emergency room visits:**	
Agree	82 (41.0)
Do not agree	118 (59.0)
**At what times would you call the physician?**	
Only at appointed hours	62 (35.2)
Only during daytime hours (excepting Saturdays and holidays)	69 (39.2)
At all hours including nights, Saturdays and holidays	45 (25.6)
**Under which circumstance would you call your physician?**	
Only in emergencies	105 (59.7)
Whenever I think I need a medical consultation	71 (40.3)
**Getting your physician’s cell phone number could interfere with his/her privacy when they’re not working:**	
Agree	145 (72.5)
Do not agree	55 (27.5)
**The physician should not be called because there are telephone centers that are active after clinic hours:**	
Agree	161 (80.5)
Do not agree	39 (19.5)
**The physician should not be called because in emergencies one can call for an ambulance or go to the emergency room:**	
Agree	168 (84.0)
Do not agree	32 (16.0)
**The physician should not be called because medical errors can occur if a physical examination is not performed:**	
Agree	168 (84.0)
Do not agree	32 (16.0)
**The physician should not be called because there is a risk of miscommunication:**	
Agree	173 (86.5)
Do not agree	27 (13.5)
**The physician should not be called because it can interfere with his clinic work:**	
Agree	148 (74.0)
Do not agree	52 (26.0)
**There is no reason against getting the physician’s personal cell phone number:**	
Agree	19 (9.5)
Do not agree	181 (90.5)
**If the HMO provided the physician with a cell phone and paid for it, that would motivate him/her to provide the cell phone number:**	
Agree	128 (64.0)
Do not agree	72 (36.0)
**If the HMO gave the physician extra pay for cell phone consultations, it would motivate him/her to provide the cell phone number:**	
Agree	151 (75.5)
Do not agree	49 (24.5)
**If the HMO gave the physician dedicated time for cell phone consultations, it would motivate him/her to provide the cell phone number:**	
Agree	151 (75.5)
Do not agree	49 (24.5)
**Have you asked for your physician’s cell phone number in the past?**	
**Yes**	28 (14.0)
**No**	172 (86.0)
**Do you have your physician’s cell phone number?**	
**Yes**	25 (12.5)
**No**	175 (87.5)

One hundred seventy-six patients (88%) said that getting their physician’s cell phone number could improve their relationship with their physician and their personal sense of security even if they did not actually use it. Eighty-two (41%) thought that getting their physician’s cell phone number might cut down on the number of emergency room visits. One hundred five (59.7%) declared that they would only contact their physician by phone in the case of an emergency, compared with 71 (40.3%) who said that they would contact their physician by phone to consult on any issue that they felt a need to consult on. In reality, only 25 (12.5%) actually had their physician’s cell phone number.

### Attitudes towards e-mail consultations with physicians

One hundred seventy-one patients (85.5%) use e-mail. Of these, 25 (14.6%) would be very interested in getting their physician’s e-mail address, 85 (49.7%) would not object to getting it, and 61 (35.7%) would not be interested in getting it (Table 3).

**Table 3:** Attitudes to medical consultation e-mail

**Question**	**N (%)**
**Do you use e-mail?**	
Yes	171 (85.5)
No	29 (14.5)
**How do you feel about getting your physician’s e-mail address?**	
Very interested	25 (14.6)
Would not object	85 (49.7)
Not interested	61 (35.7)
**Getting my physician’s e-mail address could improve the relationship between us:**	
Agree	121 (70.8)
Do not agree	50 (29.2)
**Getting my physician’s e-mail address could improve my sense of security even if I don’t use it:**	
Agree	97 (56.7)
Do not agree	74 (43.3)
**Email is an effective means of communication that could solve my problems:**	
Agree	44 (25.7)
Do not agree	127 (74.3)
**e-mail can cut down on the number of clinic visits:**	
Agree	46 (26.9)
Do not agree	125 (73.1)
**e-mail can reduce the number of emergency room visits:**	
Agree	30 (17.5)
Do not agree	141 (82.5)
**Getting your physician’s email address could interfere with his/her privacy when they’re not working:**	
Agree	98 (57.6)
Do not agree	72 (42.4)
**The physician should not be sent an e-mail because there are telephone centers that are active after clinic hours:**	
Agree	143 (84.1)
Do not agree	27 (15.9)
**The physician should not be sent an e-mail because in emergencies one can call for an ambulance or go to the emergency room:**	
Agree	159 (93.5)
Do not agree	11 (6.5)
**The physician should not be sent an e-mail because medical errors can occur if a physical examination is not performed:**	
Agree	155 (91.2)
Do not agree	15 (8.8)
**The physician should not be sent an e-mail because there is a risk of miscommunication:**	
Agree	154 (90.6)
Do not agree	16 (9.4)
**The physician should not be sent an e-mail because it can interfere with his clinic work:**	
Agree	119 (70.0)
Do not agree	51 (30.0)
**There is no reason against getting the physician’s personal e-mail address:**	
Agree	10 (5.9)
Do not agree	160 (94.1)
**At what times would you send the doctor and e-mail message?**	
Only at appointed hours	53 (48.6)
Only during daytime hours (excepting Saturdays and holidays)	30 (27.5)
At all hours including nights, Saturdays and holidays	26 (23.9)
**Under which circumstance would you send your physician an e-mail message?**	
Only in emergencies	62 (56.9)
Whenever I think I need a medical consultation	47 (43.1)
**If the HMO provided the physician with a laptop computer and paid for Internet services, that would motivate him/her to provide the e-mail address:**	
Agree	102 (59.6)
Do not agree	60 (40.4)
**If the HMO gave the physician extra pay for email consultations, it would motivate him/her to provide the email address:**	
Agree	116 (67.8)
Do not agree	55 (32.2)
**If the HMO gave the physician dedicated time for e-mail consultations, it would motivate him/her to provide the e-mail address:**	
Agree	144 (84.2)
Do not agree	27 (15.8)
**Have you asked for your physician’s e-mail address in the past?**	
**Yes**	1 (0.6)
**No**	170 (99.4)
**Do you have your physician’s e-mail address?**	
**Yes**	0 (0)
**No**	171 (100.0)

One hundred twenty-one (70.8%) said that getting their physician’s e-mail address could improve their relationship with their physician. Only 44 (25.7%) thought that e-mail was a useful tool for solving patient problems. Sixty-two (56.9%) of the patients who were interested in getting their physician’s e-mail address (109 patients) declared that they would only contact their physician by e-mail in the case of an emergency. Only one declared that he/she had requested their physician’s e-mail address and none actually had it.

### Comparison of attitudes towards getting the cell phone number or e-mail address of their physician

Most of the patients would prefer to get their physician’s cell phone number over their e-mail address (*p* < 0.0001). More of them also felt that having their physician’s cell phone number would improve the patient-physician relationship and increase their feeling of security more than getting the e-mail address (*p* < 0.0001). In practice more patients had asked for the cell phone number than e-mail address, and more actually had the cell phone number rather than the e-mail address of their physician (*p* < 0.0001) (Table 4).

**Table 4:** Comparison of attitudes relating to medical consultation by cell phone or e-mail

**Question**	**Cell phone**	**e-mail**	***p***
	**N (%)**	**N (%)**	
**All participants**			
**How do you feel about getting your physician’s cell phone number or e-mail address?**			
Very interested	93 (46.5)	25 (14.6)	
Would not object	83 (41.5)	85 (49.7)	<0.0001
Not interested	24 (12.0)	61 (35.7)	
**Getting my physician’s cell phone number or e-mail address could improve the relationship between us:**			
Agree	176 (88.0)	121 (70.8)	<0.0001
Do not agree	24 (12.0)	50 (29.2)	
**Getting my physician’s cell phone number or e-mail address could improve my sense of security even if I don’t use it:**			
Agree	169 (84.0)	97 (55.7)	<0.0001
Do not agree	31 (15.0)	74 (43.3)	
**Cell phones/e-mail are an effective means of communication that could solve my problems:**			
Agree	102 (51.0)	44 (25.7)	<0.0001
Do not agree	98 (49.0)	127 (74.3)	
**Cell phones/e-mail can cut down on the number of clinic visits:**			
Agree	138 (69.0)	46 (26.9)	<0.0001
Do not agree	62 (31.0)	125 (73.1)	
**Cell phones/e-mail can reduce the number of emergency room visits:**			
Cell phone provided by my employer	82 (41.0)	30 (17.5)	<0.0001
Extra pay for the service	118 (59.0)	141 (82.5)	
**At what times would you call or e-mail your doctor?**			
Only at appointed hours	62 (35.2)	53 (48.6)	
Only during daytime hours (excepting Saturdays and holidays)	69 (39.2)	30 (27.5)	0.057
At all hours including nights, Saturdays and holidays	45 (25.6)	26 (23.9)	
**Under which circumstance would you call or e-mail your doctor?**			
Only in emergencies	105 (59.7)	62(56.9)	0.64
Whenever I think I need a medical consultation	71 (40.3)	47 (43.1)	
**Getting your physician’s cell phone number or e-mail address could interfere with his/her privacy when they’re not working:**			
Agree	145 (72.5)	98 (57.6)	0.002
Do not agree	55 (27.5)	72 (42.4)	
**The physician should not be called or sent an e-mail because there are telephone centers that are active after clinic hours:**			
Agree	161 (80.5)	143 (84.1)	0.37
Do not agree	39 (19.5)	27 (15.9)	
**The physician should not be called or sent an e-mail because in emergencies one can call for an ambulance or go to the emergency room:**			
Agree	168 (84.0)	155 (93.5)	0.004
Do not agree	32 (16.0)	11 (6.5)	
**The physician should not be called or sent an email because medical errors can occur if a physical examination is not performed:**			
Agree	168 (84.0)	155 (91.2)	0.038
Do not agree	32 (16.0)	15 (8.8)	
**The physician should not be called or sent an e-mail because there is a risk of miscommunication:**			
Agree	173 (86.5)	154 (90.6)	0.221
Do not agree	27 (13.5)	16 (9.4)	
**The physician should not be called or sent an e-mail because it can interfere with his clinic work:**			
Agree	148 (74.0)	119 (70.0)	0.392
Do not agree	52 (26.0)	51 (30.0)	
**There is no reason against getting the physician’s personal cell phone number or e-mail address:**			
Agree	19 (9.5)	10 (5.9)	0.196
Do not agree	181 (90.5)	160 (94.1)	
**If the HMO provided the physician with a cell phone and covered the cost or a laptop computer and paid for Internet services, that would motivate him/her to provide the email address:**			
Agree	128 (64.0)	102 (59.6)	0.389
Do not agree	72 (36.0)	69 (40.4)	
**If the HMO gave the physician extra pay for cell phone or e-mail consultations, it would motivate him/her to provide the cell phone number:**			
Agree	151 (75.5)	116 (67.8)	0.101
Do not agree	49 (24.5)	55 (32.2)	
**If the HMO gave the physician dedicated time for cell phone or e-mail consultations, it would motivate him/her to provide the e-mail address:**			
Agree	172 (86.0)	144 (84.2)	0.628
Do not agree	28 (14.0)	27 (15.8)	
**Have you asked for your physician’s cell phone number or e-mail address in the past?**			
**Yes**	28 (14.0)	1 (0.6)	<0.0001
**No**	172 (86.0)	170 (99.4)	
**Do you have your physician’s cellphone number or e-mail address?**			
**Yes**	25 (12.5)	0 (0)	<0.0001
**No**	175 (87.5)	171 (100.0)	

### Comparison of sociodemographic characteristics of patients regarding their level of interest in getting their physician’s cell phone number and e-mail address

Females compared to males would be more interested in getting their physician’s cell phone number (*p* < 0.0001) (Table 5), but no differences were found regarding getting their physician’s e-mail address (Table 6). Older patients would be more interested in getting their physician’s cell phone number, and less interested in getting their physician’s e-mail address compared to younger patients (*p* < 0.0001). The highest educated patients would not object to getting their physician’s cell phone number, and would be more interested in getting their physician’s e-mail address (*p* < 0.0001). Patients suffering from chronic diseases would be more interested in getting their physician’s cell phone number and e-mail address (*p* < 0.0001).

**Table 5:** Comparison of socio-demographic characteristics of patients regarding their level of interest in getting their physician’s cell phone number

**Variable**	**Very interested N (%)**	**Does not object N (%)**	**Not interested N (%)**	***p***
**Gender**				
Male	29 (31.2)	43 (51.8)	18 (75.0)	<0.0001
Female	64 (68.8)	40 (48.2)	6 (25.0)	
**Age**				
Mean ± SD	54.5 ± 17.3	42.0 ± 14.0	32.2 ± 7.7	<0.0001
Range (years)	22-89	20-74	23-55	
**Family status**				
Married	39 (41.9)	40 (48.2)	6 (25.0)	0.127
Other	54 (58.1)	43 (51.8)	18 (75.0)	
**Country of birth**				
Israel	27 (29.0)	35 (42.2)	17 (70.8)	0.001
Other	66 (71.0)	48 (57.8)	7 (29.2)	
**Education (years)**				
Mean ± SD	9.7 ± 3.7	13.2 ± 2.2	12.8 ± 3.3	<0.0001
Range (years)	2–16	8–18	1–16	
**Employment**				
Employed	41 (44.1)	67 (80.7)	18 (75.0)	<0.0001
Unemployed	52 (55.9)	16 (19.3)	6 (25.0)	
**Health status**				
Excellent	22 (23.7)	49 (59.0)	23 (95.8)	
Good	44 (47.3)	34 (41.0)	1 (4.2)	<0.0001
Poor	27 (29.0)	0	0	
**Chronic illness**				
Yes	57 (61.3)	18 (21.7)	1 (4.2)	<0.0001
No	36 (38.7)	65 (78.3)	23 (95.8)	

**Table 6:** Comparison of socio-demographic characteristics of patients regarding their level of interest in getting their physician’s e-mail address

**Variable**	**Very interested N (%)**	**Does not object N (%)**	**Not interested N (%)**	***p***
**Gender**				
Male	12 (48.0)	41 (48.2)	26 (42.6)	0.783
Female	13 (52.0)	44 (51.8)	35 (57.4)	
**Age**				
Mean ± SD	36.0 ± 12.0	40.1 ± 12.3	47.5 ± 14.2	<0.0001
Range (years)	21–59	21–69	20–75	
**Family status**				
Married	14 (56.0)	41 (48.2)	27 (44.3)	0.611
Other	44 (44.0)	44 (51.8)	34 (55.7)	
**Country of birth**				
Israel	11 (44.0)	43 (50.6)	23 (37.7)	0.302
Other	14 (56.0)	42 (49.4)	38 (62.3)	
**Education (years)**				
Mean ± SD	14.0 ± 2.0	12.7 ± 2.4	11.3 ± 3.0	<0.0001
Range (years)	11–18	6–17	1–16	
**Employment**				
Employed	17 (68.0)	69 (81.2)	40 (56.6)	0.084
Unemployed	8 (32.0)	16 (18.8)	21 (34.4)	
**Health status**				
Excellent	20 (80.0)	49 (57.6)	25 (41.0)	
Good	5 (20.0)	36 (42.4)	28 (45.9)	<0.0001
Poor	0	0	8 (13.1)	
**Chronic illness**				
Yes	4 (16.0)	16 (18.8)	27 (44.3)	0.001
No	21 (84.0)	69 (81.2)	34 (55.7)	

## Discussion

In this study we assessed the attitudes and practice of patients in relation to obtaining and using their physician’s cell phone number or e-mail address for medical consultations. The results of the study show that most of the participating patients preferred to consult by cell phone rather than through e-mail. The number interested in phone consultations was not only higher than the level of interest in e-mail consultation, but was also substantial in an absolute sense, as almost half were “very interested” in obtaining their physician’s cell phone number. These results are similar to those that we reported previously from a parallel study in which physicians comprised the study population [20]. In that study the physicians preferred to provide patients with their cell phone numbers over their e-mail address.

There is an important difference between phone and e-mail in that phone calls disturb a consultation or other activities of the physician and e-mails can be answered when convenient. However, in phone calls the physician can pose direct questions to clarify the health problem, while by e-mail another message has to be sent and answered to accomplish this.

In a previous study conducted in a medical center (not a community setting) in Pennsylvania on the provision of cell phone numbers: a) patients believed that this act shows a greater concern for them on the part of the physician, and b) patients make good, appropriate use of this option when they needed it [21]. This form of communication is perceived as a means to improve the quality of patient care and as a means to receive feedback from patients in a way that is beneficial to physicians themselves [22].

In general, patients are happy when they have the opportunity to contact their physician by cell phone [1,11]. In the present study 86% of the patients agreed that the physicians’ employers should budget time for cell phone consultations and 75.5% stated that physicians should be reimbursed for this time. These findings are consistent with those of our previous study in which 70% of the physicians thought that they should be reimbursed for this service and 50% thought that dedicated time should be set aside for this service [20].

Among the reasons given for not providing cell phone numbers to patients, 77% said that in cases of medical emergency patients should go to the hospital and 65% said that it would impinge upon their privacy beyond formal work hours. Less than half of the patients were interested in getting the cell phone number and less than one-fifth in getting the e-mail address of their physician. These apparently low percentages might be explained by the large percentage of patients who were concerned that these means of communication would interfere with the physician’s free time. Thus, they might use out-of-hours services instead. An alternative explanation might be patients’ concerns that the lack of face-to-face contact could impair the quality of care might be a significant factor that limits their interest in cell phone and e-mail consultation, together with the high availability of healthcare services in Israel, which could lead patients to prefer to set an appointment and have face-to-face communication with their physician.

Providing patients with cell phone numbers and e-mail addresses can give them a greater sense of personal security even if they do not actively use them. If used, the number of unjustified emergency room visits and unnecessary primary care clinic visits can be reduced, leading to a decrease in the physician’s work burden. Indeed, in another study cell phone calls to physicians significantly reduced the number of emergency room visits [23]. Communication with physicians by e-mail was only studied at a later stage, although a report on communication by e-mail among physicians to discuss patient cases appeared over 10 years ago [19].

In an assessment of the use of e-mail with patients, physicians reported high levels of satisfaction with this form of communication. While they expressed concern about the level of confidentiality in e-mail communication, few discussed this issue with their patients [24].

In our study we found that 85.5% of the participating patients have e-mail addresses; 25 (14.6%) said they would be very interested in obtaining their physician’s e-mail address but only one patient reported requesting their physician’s e-mail address! In our previous study, among the reasons given by the physicians for not providing e-mail addresses to patients, 65% said that this would prevent physical examination of patients and lead to medical errors, 58% said that in cases of medical emergency patients should go to the hospital, and 57% said that e-mail communication could lead to miscommunication and litigation for medical negligence [20].

Today, millions of people around the world have access to the Internet and make increasing use of this technology. Medical consultation by way of e-mail can play a central role in healthcare, but we still lack sufficient evidence to support its use in medical care and to clarify how to use it in routine clinical practice [13].

e-mail consultation raises privacy issues. In northern European countries consultation by general e-mail is officially forbidden because patient privacy cannot be guaranteed. As a result, hospitals have developed patient portals through which patients can consult with their physicians [2,25]. In Israel there are no ethical or legal guidelines or rules on the provision of medical consultation by e-mail or cell phone, or for social networks such as Facebook and Twitter.

Together with the advantages cited above there are also limitations to the use of this technology such as the intrusion into physicians’ privacy during off-work hours, interruption during other patients’ clinic visits, and the danger of miscommunication and medical error [26]. Allocating resources, such as work time or reimbursement for these services could improve physician compliance [27]. The integration of nurses into cell phone or e-mail consultations could improve medical care for patients and would be a more affordable solution since a nurse’s time is less expensive than a physician’s and the response time could be shortened.

This study has several limitations. It was conducted in one geographic region in southern Israel so the results cannot necessarily be generalized to other areas in Israel or the rest of the world. It is possible that Israeli patients, or at least patients from the study region, are less Internet savvy than their counterparts in the U.S. and northern Europe. The percentage of patients interested in e-mail communication with their physician was relatively low in the present study, but we do not have precise data on Internet use in the region. The interviews were conducted only with patients who agreed to participate in the study and who could provide the required information. Those who could not be interviewed or did not agree to be interviewed may represent a different population, possibly from a lower socio-economic stratum, who may be less interested in conducting cell phone or e-mail consultations. This difference could lead to biased results.

## Conclusions

The patients interviewed favored use of cell phones to e-mail for communication with their physicians and for medical consultation. As new technologies such as cell phones and e-mail become more available and widely used, it is important to understand the significance of integrating these means into clinical practice as well as how that integration should be accomplished. The allocation of time and/or payment to the physician for e-mail or cell phone (with a dedicated phone number) consultations could improve non–face-to-face healthcare service to patients. There is often strong resistance on the part of physicians to this type of non-frontal care. The formulation of medico-legal guidelines and rules and appropriate accreditation could make non-frontal service more acceptable to them. We hope that the study results will help to integrate these new technologies into clinical practice for the improvement of the quality of patient care.

While this study focused on medical consultations by means of cell phones and e-mail, future studies should be nationwide in Israel including patients who speak only Arabic, and they should also investigate the potential contribution of social networks such as Facebook and Twitter.

## Competing interests

The authors declare that they have no competing interests.

## Authors’ contributions

RP designed the study, and in cooperation with EN was responsible for writing the research proposal. EN managed the data collection, both authors analyzed and interpreted the data, and participated in manuscript writing and editing together. Both authors read and approved the final version of the manuscript.

## Authors’ information

Roni Peleg is director of the Wingate Clinic of the Clalit Health Services, Beer-Sheva, Israel, and an Associate Professor in Family Medicine at the Faculty of Health Sciences, Ben-Gurion University of the Negev, Beer-Sheva.

Elena Nazarenko is a family physician in the “E” clinic of the Clalit Health Services, Beer-Sheva, Israel.
